# Progress on the research and development of plague vaccines with a call to action

**DOI:** 10.1038/s41541-024-00958-1

**Published:** 2024-09-07

**Authors:** E. Diane Williamson, Paul B. Kilgore, Emily K. Hendrix, Blake H. Neil, Jian Sha, Ashok K. Chopra

**Affiliations:** 1 Defence Science and Technology Laboratory, Porton Down, Salisbury, SP4 0JQ UK; 2 Department of Microbiology and Immunology, UTMB, Galveston, TX 77555 USA; 3 Sealy Institute for Vaccine Sciences, UTMB, Galveston, TX 77555 USA; 4 Institute for Human Infections and Immunity, UTMB, Galveston, TX 77555 USA; 5 Center for Biodefense and Emerging Infectious Diseases, UTMB, Galveston, TX 77555 USA; 6 Galveston National Laboratory, UTMB, Galveston, TX 77555 USA

**Keywords:** Bacterial infection, Bacterial infection

## Abstract

There is a compelling demand for approved plague vaccines due to the endemicity of *Yersinia pestis* and its potential for pandemic spread. Whilst substantial progress has been made, we recommend that the global funding and health security systems should work urgently to translate some of the efficacious vaccines reviewed herein to expedite clinical development and to prevent future disastrous plague outbreaks, particularly caused by antimicrobial resistant *Y. pestis* strains.

Content includes material subject to Crown Copyright © 2024.This is an open access article under the Open Government License (http://www.nationalarchives.gov.uk/doc/open-government-licence/version/3/).

## Main text

### Epidemiology of plague

Plague, caused by the gram-negative bacterium *Yersinia pestis*, is notorious for its involvement in three of the seven deadliest pandemics recorded in global history, including the recent COVID-19 pandemic. The three historic plague pandemics, the most infamous of which was the Black Death of the Middle Ages, collectively caused an estimated 200 million deaths^1,2^. Unfortunately, plague is still an endemic disease in parts of the world, with outbreaks being reported to the WHO from over 33 countries including Madagascar, Democratic Republic of the Congo (DRC), India, China, Peru, and occasionally, the south-western USA^3^. In these regions, disease is maintained by the existence of infected animal (mostly rodent) reservoirs of *Y. pestis*^4,5^.

**Fig. 1:**
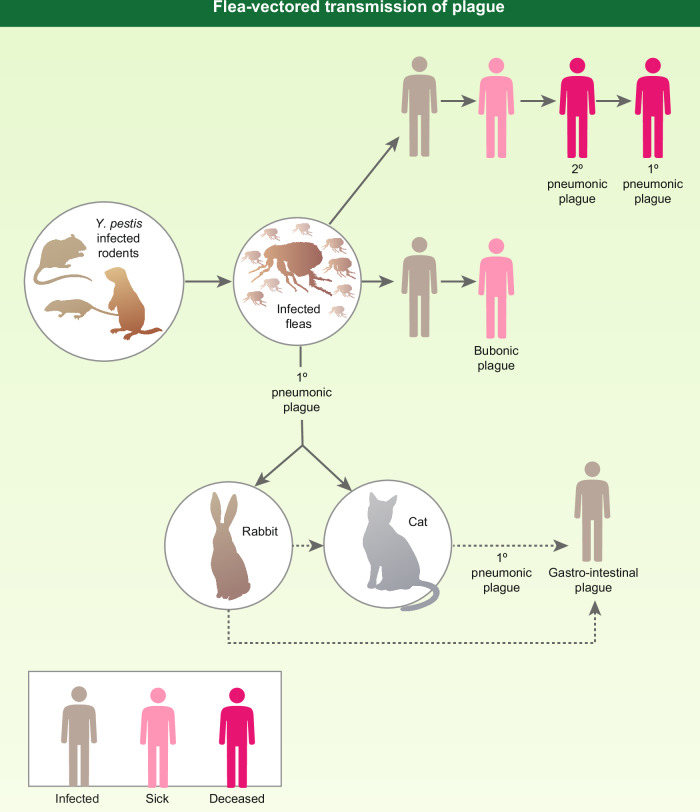
Flea-vectored transmission of plague. The figure depicts various routes for the flea-vectored transmission of plague to man. The figure is reproduced from Williamson and Westlake (2019)^8^ with permission (License 5753521304382, Oxford University Press).

Transmission to humans is predominantly promoted by flea bite; those fleas having fed on infected rodents (Fig. 1). However, *Y. pestis* is an obligate parasite and even if the rat population is reduced, the organism can infect mice, prairie dogs, rabbits, and members of the cat family, including the domestic cat^4–6^.

Infection through flea bite causes bubonic plague, which if undiagnosed, can develop into a septicemic infection or secondary pneumonic plague. Pneumonic plague is highly contagious requiring prompt antibiotic therapy for survival, as the mortality rate approaches 100% if untreated^6–8^. In pneumonic plague, *Y. pestis* can be transmitted to healthy individuals who are in close contact by respiratory droplets, establishing further cases of primary pneumonic plague, leading to disease outbreaks which may transform into epidemics and pandemics^6^.

With this epidemiology, poor living conditions augment the endemicity of plague, which requires close contact with a rodent population. However, in endemic regions such as Madagascar, the lack of an approved vaccine means that outbreaks have to be controlled by antibiotic therapy, administered to the patients and those in immediate contact with the infected individuals. Whilst timely antibiotic therapy is effective in treating the infection, case fatality rates still reached up to 8.6% during the 2017 Madagascar outbreak despite aggressive antibiotic therapy^9^. Additionally, there is a demonstrable risk of the development of antibiotic resistance. Indeed, antimicrobial resistant (AMR) *Y. pestis* strains have been identified in Peru and Madagascar^10,11^. Therefore, there is a clear need for a safe, effective, and licensed vaccine for use in endemic regions to control or prevent infection, as well as to protect military and civilians at large from potential biothreat attacks.

### Emergence of *Yersinia pestis* as a dangerous pathogen

*Y. pestis* has evolved from the relatively mild gastrointestinal *Y. pseudotuberculosis* (notably serotype 1b) between 1500 and 20,000 years ago^12^, although archaeological evidence has suggested that the plague-causing bacterium existed long before previous estimates^13^.

The evolution of *Y. pestis* has resulted in the inactivation of genes required for an enteric lifestyle and by the acquisition of plasmids encoding new virulence factor-encoding genes^14^. In common with other pathogenic yersiniae (*e.g., Y. pseudotuberculosis* and *Y. enterocolitica*), *Y. pestis* possesses a 70-kilobase (kb) virulence plasmid designated as pYV/pCD1 that carries a Type III secretion system (TTSS) operon^15,16^. However, *Y. pestis* has acquired two additional unique plasmids, including a 9.5-kb pPCP1/pPla/pPst encoding a bacterial surface-bound protease (plasminogen activator, Pla), which has potent fibrinolytic activity^1^. In addition, this plasmid possesses pesticin and coagulase encoding genes which enable bacterial transmission from the flea^17^. The other 100–110 kb pFra/pMT1 plasmid^18^ codes for two important proteins, Fraction 1 (F1) antigen and a phospholipase D known as murine toxin. The F1 antigen forms a polymeric anti-phagocytic capsule around the bacteria^18^ whilst murine toxin has a role in preserving *Y. pestis* in the flea gut^19^. During its evolution from enteric to flea-vectored pathogen, *Y. pestis* has lost intestinal adhesin and invasin genes, but has retained the heme locus and possesses a number of chromosomal-encoded genes such as the *ph6/psa* fimbrial and attachment-invasion locus (*ail*) which promote colonization to the host cells^19–21^.

### Evasion of host responses

In the process of acquiring a new mechanism of infection, *Y*. *pestis* has also activated genes which enable the pathogen to evade the defenses of its successive hosts. In purified or recombinant forms, some of these encoded gene products have provided vaccine targets and are therefore summarized here.

*Y. pestis* can survive and grow in the flea’s (most notably the rat flea *Xenopsylla cheopis*) foregut, leading to ‘blockage’ of the flea. The proper functioning of the bacterial hemin storage system is thought to play an important role in the formation of this blockage^19^, which during the flea bite, results in the regurgitation of a dense bolus of bacteria^5^ into a new host. *Y. pestis* expresses other genes in the flea gut such as a ‘murine’ toxin with phospholipase D activity^20^ and a lipopolysaccharide (LPS) core modification locus, which together are required for biofilm formation and blockage of the flea^20–22^. However, transcriptional analysis of *Y. pestis* in the flea gut has identified a wide range of additional genes, such as insecticidal-like toxin genes, which are differentially regulated such that bacteria regurgitated into a new host have increased resistance to innate immune effectors^23^.

Upon infection of a new mammalian host, the plague bacilli are vulnerable to phagocytosis by polymorphonuclear leukocytes (PMNs or neutrophils) and/or monocytes. The bacteria may be killed within PMNs, but can persist within monocytes and express various virulence determinants, allowing *Y. pestis* growth and eventual release from the monocytes^24^. The fibrillar adhesin pH6 antigen is induced by low phagosomal pH (4.5)^25^ and promotes bacterial adhesion to host cells, thereby enhancing resistance to phagocytosis^26^. Secretion of the F1 antigen with capsule formation is triggered by a temperature shift from 28 °C in the flea to 37 °C in humans or other mammals. The F1 capsule also plays a key role in avoiding phagocytosis^27^. However, non-capsulated *Y. pestis* retains its full capability to cause pneumonic infection in animals, while having reduced virulence during bubonic infection^28^.

The dominant anti-host effects are due to a temperature shift induction of the TTSS carried on the virulence plasmid pYV/pCD1. TTSS effectors, historically called *Yersinia* outer membrane proteins (Yops), have cytotoxic and phagocyte regulatory effects, are secreted through an injectosome after *Y. pestis* makes contact with the host cell, and are delivered into target cells^15^. The function of many of the Yops has been delineated for this well-characterized secretion system, and serves as a paradigm for other bacterial TTSS’s^15^. For example, the YopE protein is a cytotoxin and the YopH protein is a tyrosine phosphatase with anti-phagocytic activity^29^. The V (or Low calcium response V, LcrV) antigen plays a pivotal role by orchestrating intracellular Yop low calcium response protein G (LcrG) elaboration of the injectosome and then itself being delivered through this needle-like structure to be assembled as a pentamer at the tip^30^. Additionally, V antigen secreted from *Y. pestis* exerts a local immunomodulatory effect in the host by down-regulating the production of interferon-γ (IFNγ) and tumor necrosis factor-α (TNFα)^31,32^.

Plasminogen activator (Pla) is another major virulence factor in *Y. pestis*. Pla is an outer membrane-located protease, which breaks down the physical barriers of connective tissue in the host, thus promoting the systemic dissemination of the bolus of *Y. pestis* injected by the flea. The requirement for Pla has driven the selection in *Y. pestis* of the “rough” phenotype of LPS, which lacks an O antigen^33,34^, a rare phenomenon amongst gram-negative bacteria which has possibly resulted from the bacterium’s transmission through the flea, but which is necessary for Pla to be functional^35,36^. Inactivation of the O-antigens on *Y. pestis* LPS exposes the LPS core, so that *Y. pestis* can interact with C-type lectin receptors on host macrophages, promoting its uptake, and thus accelerating bacterial dissemination in the host^37^. Our study has also shown that the Δ*pla* mutant is unable to survive efficiently in murine and human macrophages, unlike the wild-type *Y. pestis*^38^.

The bacteria disseminate from the site of primary infection into draining regional lymph nodes. Within the lymph node, further growth of the bacteria accompanied by a massive inflammatory reaction leads to lymphadenopathy and the formation of buboes, typically in the groin or axillae. In the bubo, bacteria are predominantly extracellular, mainly due to the TTSS which is highly expressed in the lymph node^39^. An ability to proliferate in the bubo^40^ is enabled by the efficient and abundant iron acquisition systems possessed by *Y. pestis*^41^.

Eventually, the bacteria are disseminated by the lymphatic system, gain access to the blood stream, and colonize pulmonary tissues, which may lead to development of the pneumonic form of the disease. When left untreated, pneumonic plague induces an overwhelming septicemia which triggers septic shock in the host. However, the precise mechanisms that lead to the death of the host have not been identified but involve multi-organ failure, during which the systemic induction of nitric oxide synthase may contribute, as seen with other gram-negative septicemias^42^.

Whilst pigmentation (*pgm*)-negative strains of *Y. pestis* are usually avirulent and attenuated, the risk of reversion to virulence was highlighted by the fatal case of a laboratory worker who was unknowingly suffering from hemochromatosis and was exposed to the attenuated *pgm*^-^ *Y. pestis* laboratory strain KIM. This individual developed plague and died, presumably due to his hemochromatosis-induced iron overload condition providing the infecting KIM strain, attenuated through defects in its iron acquisition ability, with sufficient iron to render it virulent^43^.

### Early vaccines

The early use of an inactivated whole cell vaccine for plague by Haffkine between 1897 and 1935 successfully curtailed plague outbreaks in India. This was the first demonstration that components of *Y. pestis*, even when inactivated, could be immunogenic. Haffkine’s heat-killed whole cell (KWC) vaccine was administered to the human population in an estimated 24 million doses^44^ (Table 1).

**Table 1:** Early generation plague vaccines

Vaccine	Type	Doses	Route	Species tested	Protection	Type of Immune Response	Shortcomings	Years studied (Ref)
Haffkine vaccine	Heat-killed	1	s.c.	rabbits	Bubonic only	Likely Ab only	Severely reactogenic	1897–1935^44^
Plague vaccine (USP) Or CSL vaccine	Formalin-inactivated Heat-inactivated	3+ 3	i.m. i.m.	Mice mice	Bubonic only	Ab	Frequent boosters, reactogenic	1939–1999^45–53^
Live plague vaccine (EV76, EVNIIEG)	Live-attenuated	1+	Various $	Mice, Rats, Guinea Pigs, NHP’s*	Both bubonic and pneumonic	Ab and CMI	Frequent boosters, Reactogenic, Virulent during iron overload	1936–present^54–57^

Dollar sign indicates various routes include skin scarification, intradermal, sub-cutaneous (s.c.), oral (p.o) and inhalational. Asterisk indicates live plague vaccine can cause disease in African Green monkeys (AGMs). In humans, EV76 is recommended to be administered once a year. It is used in Former States of Soviet Union and regions where plague is endemic but is not approved in USA/Europe; antibodies to F1, LcrV and YscF have been detected in vaccinated humans. Commonwealth Serum Laboratories (CSL) in Australia produced heat-killed vaccine, administered in 3 doses in humans. *Ab* Antibody, *CMI* Cell-mediated immunity, *GP* guinea pig, *NHP* non-human primate, *s.c.* sub-cutaneous, *i.m.* intra-muscular.

During the 1990s, there were several commercial suppliers of the KWC vaccine against plague. Subsequently, plague vaccine USP (United States Pharmacopeia; 1939–1999), containing formaldehyde-killed bacteria, was manufactured by Cutter Laboratories, USA. In 1994, the manufacturing was transferred to Greer Laboratories Inc., USA. In 1999, the production of this vaccine was discontinued largely because of severe side effects and its protection against bubonic but limited efficacy against pneumonic plague^45–52^. An alternative heat-killed (KWC) vaccine was also manufactured by the Commonwealth Serum Laboratories (CSL, Australia)^53^ and until November 2005, was licensed for clinical use in Australia. Additionally, a *Y. pestis* isolate (EV76-NIIEG *Y. pestis*)^54^, which is attenuated due to deletion of the pigmentation locus (*pgm*), has been used as a vaccine for many years and is licensed for use in China and Russia specifically^55^ where plague is endemic. The vaccine can be administered by various routes; however, the vaccine is fully virulent under iron-overload conditions, *i.e*., in individuals with hemochromatosis^56,57^ (Table 1).

### Virulence factors as vaccine antigens

The seminal observation in 1956 by Bacon and Burrows that *Pasteurella pestis* (now *Y. pestis*) could be anti-phagocytic in the absence of capsule, led to the identification of a new virulence antigen, which they named the V antigen^58^. This paved the way for subsequent research to the present day on the immunogenic and protective potential of this and other virulence factors of *Y. pestis*^59,60^. Building on the observation that the F1 antigen-containing Cutter KWC vaccine needed the addition of a recombinant V (rV) antigen to fully protect mice against pneumonic plague^61^, Williamson, et al. demonstrated the synergistic effect of F1 and V in combination. Whilst vaccines lacking the V antigen may protect against bubonic plague, several groups showed that the inclusion of the V antigen was an essential requirement for protection against pneumonic plague^61,62^.

Much work has been carried out to determine the protective potential of other antigens derived from *Y. pestis* in native or recombinant form in addition to F1 and V, such as Pla, a protein constituent of the injectisome known as *Yersinia* secretory factor F (YscF), and a range of other Yops, and their various combinations^63–67^. Whilst some of these imparted partial protective efficacy and are useful adjuncts in some vaccine formulations (see below), to date F1 and V remain the key proteins which individually have protective efficacy, but which in combination, are consistently synergistic and, therefore, form the core building blocks of most vaccine approaches.

### Vaccines for plague

**Fig. 2:**
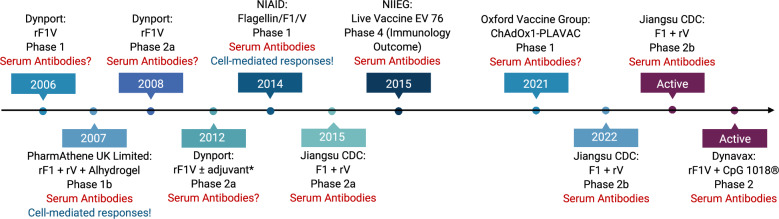
Plague vaccines in clinical trials. *Adjuvant not specified. Ages of study participants ranges from 18 to 55 years. All vaccines were given in 2–3 doses intramuscularly over a range of 6 months. The EV 76 NIIEG vaccine was given 1–4 times at intervals of 1–3 months. ?data not published; !data not conclusive.

Currently, there are more than 21 candidate vaccines in the preclinical phase^3^. Below, we have reviewed the pre-clinical candidates (Tables 1–5) and subsequently those that are in early clinical development with a timeline (Fig. 2). The pre-clinical candidates can be broadly categorised as subunit, live attenuated, vectored (bacterial or viral), DNA, or messenger RNA (mRNA).

**Table 2:** New subunit plague vaccines and adjuvants

Vaccine	Doses	Adjuvant	Route	Species tested	Efficacy*	Immune response type	Years studied (Ref)
rF1-V	2	Alum	s.c.	Mice. NHP	Pneumonic	Ab	1998–present^60^^,69,70,85^
rF1 + rV	2	Alum	i.m.	Mice, GP, NHP	Pneumonic	Ab	1995–2011^59,61,68,71^
Calcium phosphate Protein-coated microcrystals (PCMC) F1V	2	Alum	s.c.	Mice	Pneumonic	Ab	2018–2022^82^
Flagellin F1-V	2	Flagellin	i.m.	Mice NHP ^$^	Pneumonic	Ab	2006–2020^74,75,124^
Protollin F1-V**	2	Protollin	i.n.	Mice	Pneumonic	Ab	2006^73^
Single dose F1-V Polyanhydride nanoparticles coupled with cyclic dinucleotides	1	STING (stimulator of interferon genes) agonist	i.n.	Mice	Pneumonic	Ab and cell-mediated (CMI)	2019^76^
rV10	2	alum	i.m.	Mice, GP, NHP	Pneumonic	Ab	2005–2011^71^
Peptidoglycan-free OMV (Bacterial ghosts)-phage lytic system	2	self	s.c.	Mice GP	Bubonic	Ab and CMI	2021^78^
Manganese silicate nanoparticle rF1-V10	2	self	s.c.	Mice	Pneumonic	Ab and CMI	2023^77^
Polymeric F1 + LcrV (ILB1)-R	1	alum	s.c.	Mice	Pneumonic	Ab	2023^72^
*Y. pseudotuberculosis*-based LcrV MPLA OMV	2	MPLA (monophosphoryl lipid A)	i.m.	Mice	Pneumonic	Ab and CMI	2020–2023^81,105^
Plague microencapsulated vaccine (licensed in Russia)	2	Alum + self	s.c.	Mice GP NHP Humans	Bubonic	Ab and CMI	1983–2018^86^

Asterisk indicates pneumonic infection can be *via* aerosol or intra-nasal. Double asterisk indicates proteosomes are non-covalently coupled to LPS. Dollar sign indicates no challenge data shown. *GP* Guinea pig, *NHP* Non-human primate, *OMV* outer membrane vesicles, *Ab* Antibody, *CMI* Cell-mediated immunity, *s.c.* sub-cutaneous, *i.m.* intra-muscular, *i.n.* intra-nasal.

**Table 3:** New generation live- attenuated plague vaccines

Vaccine	Doses	Mutation	Route	Species tested	Safety shown in immuno-compromised models	Efficacy	Type of immune response	Years studied (Ref)
Y*. pestis* CO92 ΔLMA*	1–2	*lpp, msbB, ail*	i.n. or i.m.	Mice, rats	Rag1 KOO/iron overload**	pneumonic	Ab and CMI	2015^88–91^
*Y. pestis* CO92 ΔLMP	1–2	*lpp, msbB, pla*	i.m.	Mice, rats	safe	pneumonic	Ab and CMI	2016^88^
*Y. pestis* EV76-B-SHU Δpla	3	*pgm, pla*	i.t. or s.c.	mice	Not tested	pneumonic	Ab and CMI	2020^92^
*Y. pestis* CO92 ΔpgmΔpPst	1–2	*pPgm, pPst (pla)*	s.c.	mice	Not tested	pneumonic	Ab and CMI	2021^93^
*Y. pestis* CO92 ΔyscN	1–2	*yscN*	s.c.	mice	Not tested	Bubonic and pneumonic	Ab and CMI	2021^93^

Single asterisk indicates no clinical symptoms observed in cynomolgus macaques or African Green monkeys (unpublished), double asterisk indicates avirulent under iron overload conditions. *Ab* Antibody, *CMI* Cell-mediated immunity, *s.c.* sub-cutaneous, *i.m.* intra-muscular, *i.n.* intra-nasal, *i.t.* intra-tracheal.

**Table 4:** DNA and bacterial and viral-based, as well as mRNA-based plague vaccines

Vaccine	Type	Doses	Route	Species tested	Efficacy	Immune response	Years studied (Ref)
DNA F1-V	DNA vaccine	Up to 6	i.m.	Mice	pneumonic	Ab & CMI	1999–2012^45,119^
Ad5-F1 + Ad5-LcrV	Adenoviral vector	2	i.m.	Mice	pneumonic	both	2006–2010^116,118^
Ad5-YFV	Adenoviral vector	2	i.n.	Mice, NHP	pneumonic	both	2016–2023^113,114^
T4-phage	Prokaryotic viral vector	2	i.m.	Mice, rats	pneumonic	both	2013–2023^112^
*S*. Typhimurium expressing plague antigens	Bacterial vector	1–2	Mostly p.o.	mice	pneumonic	both	1996–2016^107–109^
*S*. Typhi expressing plague antigens	Bacterial vector	1–3	i.n.	mice	Bubonic, septicaemic	both	2004–2009^106^
*Lactiplantibacillus plantorum* expressing lcrV	Bacterial vector	3*	p.o.	mice	Not tested	both	2011^45^
F1-mRNA-LNP	mRNA-LNP	1	i.m.	mice	bubonic	both	2023^120^
*Y. pseudotuberculosis* producing F1	Bacterial vector	1+	s.c or p.o.	mice	Bubonic, pneumonic	both	2008–2020^101–104^
Self-amplifying mRNA(F1 + lcrV)	mRNA-LNP	2	i.m.	mice	bubonic	both	2023^121^
*F. tularensis*Δ*capB* + F1-LcrV/PA	Bacterial vector	2	i.m., i.n.	mice	Respiratory infection	both	2018^111^

Asterisk indicates that each dose consisted of 2x daily administrations for 3–4 days. *Ab* Antibody, *CMI* Cell-mediated immunity, *s.c*. sub-cutaneous, *i.m.* intra-muscular, *i.n.* intra-nasal, *p.o.* oral.

**Table 5:** Plague vaccines tested in NHP’s or heterologous vaccination strategy

Vaccine	Type	Adjuvant	Doses	Route	Cyno macaque efficacy	African Green monkey efficacy	Type of immune response	Years studied (Ref)
rF1-V	Subunit	alum	3	s.c.	80%	20%	Ab	2007–2018^51^
LicKM-LcrV-F1	Subunit	LickM + alum	3	s.c.	100%	Not tested	Ab	2007–2009^130^
rF1 + rV	Subunit	alum	2	i.m.	100%	Not tested	Ab	2011^68^
rV10	Subunit	alum	3	i.m.	100%*	33%	Ab	2011^71^
rAd5-YFV + rY FV $	Viral vector with protein boost	self	1 + 1	i.n.-i.m.	100%	Not tested	Ab	2016^114^
Microvesicle *Bacteroides* spp.) F1-V	OMV	self	2	p.o./i.n.	Not tested	Not tested	Robust IgA and IgG in blood and airways	2019^80^
**Heterologous prime-boost**								
**Vaccine**	**Type**	**Adjuvant**	**Doses**	**Route**	**Efficacy in mice**		**Type of immune response**	**Years Studied (Ref)**
Ad5-YFV/LMA**	Hetero-logous	self	1 + 1	Both i.n.	Pneumonic & bubonic		Ab and CMI	2021–2023^56^

Single asterisk indicates that only 50% of controls died. Double asterisk indicates that no clinical signs were observed in cynomolgus macaques or in African green monkeys; dollar sign indicates that Ad5 pre-existing immunity was induced prior to immunisation. *OMV* outer membrane vesicles, *Ad5-YFV/LMA* Ad-vectored and live attenuated, *Ab* Antibody, *CMI* cell-mediated immunity, *s.c.* sub-cutaneous, *i.m.* intra-muscular, *i.n.* intra-nasal, *p.o.* oral.

#### Subunit

Many groups have now shown that immunization with the F1 and V subunit antigens provides a high degree of protection against infection caused by *Y. pestis* in a range of animal models^68–78^. The use of F1 and V in combination (F1 + V) or as a genetic fusion (F1-V) has the advantage that protection can be maintained against acapsular (F1-negative) *Y. pestis* strains which still retain virulence^79^. Many different formulations have been researched with a view to finding one that provides comprehensive protection with the least number of doses, is stable, and maintains immunogenicity when escalated up the species from mice to humans. These candidate vaccines which have been studied in much more detail are summarized in Table 2.

Adjuvants used in preclinical rF1V vaccine development studies include the toll-like receptor 5 (TLR5) ligand flagellin and protollin^73–75^. Packaged in a polyanhydride nanoparticle with cyclic dinucleotide and delivered as a single dose intranasally, rF1V protected mice against pneumonic plague^76^. A truncated form of rV (rV10) has also been shown to be protective, as has rV10 in manganese silicate nanoparticles^71,77^. Similarly, the peptidoglycan-free outer membrane vesicles (OMV) with a phage lytic system has been demonstrated to be efficacious^78^, as have microvesicles derived from human commensal gut bacteria for immunization with V antigen^80^ or OMV’s from *Y. pseudotuberculosis*^81^. A dry formulation of rF1+rV delivered on calcium phosphate-decorated microparticles demonstrated enhanced immunogenicity and efficacy^82^.

A study has shown that the polymeric form of F1 led to rapid protective humoral immune response by activating innate-like B1b cells^83^ (Table 2), and further observations suggested that this activation was unaffected by the presence of the V antigen in an admixture of F1 and V^83^. Recent research has evaluated the impact of the administration of synthetic immunomodulating peptides on the survival of mice and guinea pigs subsequently exposed to virulent *Y. pestis*^84^. Administered in three doses prior to animal challenge, two immunomodulators were found to have a positive impact on survival; these were an azoximer bromide (polyoxidonium) and rIFNγ^84^. Another study has shown that co-formulation of the rF1-V vaccine with recombinant human (rhIL2) and/or recombinant murine GM-CSF in alhydrogel enhanced immunogenicity and efficacy against a lethal aerosol challenge in mice^85^.

In April 2024, the Russian state regulator granted a marketing authorization^86^ for a single sub-cutaneous dose microencapsulated molecular plague vaccine (PMMM) comprising 25–30 µg each of rF1 and rV in 4–6 mg polylactide, with the excipients polyvinyl alcohol, alhydrogel, polyvinyl pyrrolidine, polysorbate in phosphate buffered saline and containing 30–60 µg thiomersal.

#### Live attenuated

The live attenuated vaccine (LAV) strain EV76-NIIEG has been used for human vaccination in Russia and China for many years to prevent or curtail outbreaks of plague^3^. Recently, the experimental evaluation of polyoxidonium co-administered with EV76 in a murine model has been shown to improve efficacy^87^.

In addition to the live vaccine strain EV76, various deletion mutants of *Y. pestis* CO92 have been demonstrated to be efficacious in rodent models of pneumonic plague^88–91^ (Table 3). Among these, LMA and LMP mutants (deleted for genes encoding Braun lipoprotein [Lpp], methylacyl transferase B [MsbB], and either Attachment-invasion locus [Ail] or Plasminogen-activating protease [Pla], were of note, as they triggered robust humoral and cell-mediated immune responses in mice and were eliminated from the animals within 12–24 h^88–91^. Importantly, these mutants remained avirulent under iron-overload conditions^56^. Further, a heterologous prime-boost strategy using one dose each of LMA or LMP and replication-defective adenovius5-based three component vaccine containing genes for YscF, F1, and LcrV (Ad5-YFV) administered in any order was highly efficacious with complete protection in mice in a pneumonic plague model^56^ providing safety and combined benefits of subunit and live-attenuated vaccines (Table 5). Likewise, EV76 vaccine deleted for Pla^92^ has shown promise. Recent work has also addressed the possibility of further attenuation of *Y. pestis* to serve as a vaccine^93^. The two most protective vaccine candidates were *Y. pestis* CO92 mutants that were either cured for the *pgm* locus and the pPst plasmid or deleted for the *yscN* gene. These mutants completely protected BALB/c mice against subcutaneous and aerosol challenge with *Y. pestis*^93–95^ (Table 3).

#### Vectored

Since the potential to harness rDNA technology to produce vaccines in the 1980s, many more candidate plague vaccines have been pursued^45,46,52,96–116^, including the use of attenuated bacterial or viral vectors to deliver antigens derived from *Y. pestis*. Vectors being evaluated include: *Salmonella*, *Yersinia pseudotuberculosis*, *Lactobacillus*, adenovirus, vesicular stomatitis virus, and vaccinia virus. Some of these well-studied vaccine candidates have been summarized in Table 4.

A common advantage of these vectors is that because they are live, but replication-deficient, only 1 or 2 doses of vaccine may be required to achieve protective immunity. A second advantage is that these vectored vaccines can be multivalent, expressing antigens from different pathogens and can deliver these antigens intracellularly, mimicking infection and inducing appropriate immunity. All of these vaccine vectors require an in vivo promoter to switch on the expression of a heterogenous antigen(s) to induce an immune response. The efficiency of the promoter and the molecular size of the expressed protein-encoding genes, together with need for post-transcriptional modifications such as glycosylation, determine the level and potency of the expressed vaccine antigens. Potential disadvantages of live vaccine vectors are the necessity of stable attenuation, the risk of use in immunocompromised individuals, the possibility of inducing immunity, or pre-existing immunity to the vector itself; however, the latter can be overcome by modification of the vector or by employing a heterologous prime-boost approach to prevent reduced responses on repeated use of the same vector^99,100^ as we have recently shown^52^. Further, our study in non-human primates showed that inducing pre-existing antibodies to Ad5 did not alter protective immune responses in a pneumonic plague model^114^.

#### Bacterial

A substantial amount of research has been devoted to the development of *Y. pseudotuberculosis* as a vaccine for plague by deleting three essential virulence factors (High Pathogenicity Island, pH6 antigen, and YopK toxin) and by the insertion of the *caf* operon into the chromosome, allowing the production of an F1 pseudocapsule^101–104^. A *Y. pseudotuberculosis* construct (VTnF1) modified to maximize stability was immunogenic and efficacious against pneumonic plague in mice after a single oral dose^102^, and generated humoral and cell-mediated immune responses^103^. Subsequently, the VTnF1 vaccine has been shown to be effective in mice after subcutaneous injection and protects fully against injected (10^4^ LD_50_) or 80% of animals against aerosolized (3300 LD_50_) *Y. pestis* CO92^104^ (Table 4).

Likewise, outer membrane vesicles (OMV) produced from a mutated version of *Y. pseudotuberculosis* expressing V and a modified version of LPS have been shown to be protective in mice against pneumonic plague^105^ (Table 4).

There has also been substantial research investment in *Salmonella* Typhi as a vaccine vector, particularly with its potential as an oral vaccine for plague. Early studies showed that the successful carriage by *S*. Typhimurium of the F1-encoding plasmid resulted in F1 protein secretion by *S*. Typhimurium, with visualization of the capsule surrounding the bacteria^106^. However, whilst immunogenicity and efficacy were achieved, sustaining the vector in vivo to retain plasmids with sufficient gene expression over time without causing salmonellosis, has been an enduring challenge^107,108^. More recently, combinations of F1, Psn (pesticin receptor), and V antigen delivered orally to mice using mutant strains of *S*. Typhimurium have provided 100% protection against subcutaneous challenge with 570 LD_50_ of *Y. pestis* CO92, but only 40-60% efficacy against 50 LD_50_ of aerosolized *Y. pestis* CO92^109^. Moreover, *S*. Typhimurium deleted for the genes *lpp* and *msbB* and used to express F1, LcrV, a combination of F1 and LcrV, and a combination of YscF and YopD, protected mice against *Y. pestis* CO92 infection in a pneumonic plague model^45,110^.

Expression of genes encoding F1 and LcrV of *Y. pestis* and protective antigen (PA) of *Bacillus anthracis* in a *Francisella tularensis* LVS vaccine strain provided protection to mice against all three Tier-1 select agents, raising the prospect of a polyvalent biodefense vaccine^111^ (Table 4).

Bacteriophage T4 serves as an excellent nanoparticle platform to deliver plague immunogens (F1 and V as well as PA antigen of *B. anthracis*). Both mice and rats immunized with T4 phages without any adjuvant and harboring *Y. pestis* and *B. anthracis* immunogens were protective against pneumonic plague and lethal toxin intoxication when administered sequentially or simultaneously^112^ (Table 4).

#### Viral

Recent preclinical studies have shown that vaccination of mice with Ad5-YFV provided complete protection to mice in a pneumonic plague model when challenge occurred with the F1-minus strain of *Y. pestis* CO92^113^. This is when compared to animals that were vaccinated with the monovalent, Ad5-LcrV-based vaccine, and challenged with the F1-minus strain of *Y. pestis* CO92 where anti-F1-antibodies were rendered ineffective^113^. This Ad5-YFV vaccine resulted in robust humoral and cell-mediated immune responses^114^. The above vaccine also provided 100% protection to Cynomolgus macaques at a very high challenge dose of *Y. pestis* CO92 administered by the aerosol route^114^ (Tables 4 and 5). An earlier version of Ad5-based vaccine harbored genes for F1 and LcrV and was shown to be protective in a murine pneumonic plague model^115,116^.

A chimpanzee adenovirus vector (ChAdOX1) vaccine expressing F1 and V has been developed by the Oxford Vaccine Group. The ChAdOX1 vector is a replication-deficient adenoviral vector based on the simian adenovirus type Y25, originally chosen to avoid pre-existing adenovirus immunity in the human population^117^. The phase 1 clinical trial started on this ChAdOX1 plague vaccine in 2021^118^ (Fig. 2).

#### DNA

DNA-based plague vaccines comprising F1 and LcrV have also been tested and found to be immunogenic and protective (Table 4). In an earlier study, we have shown that mice immunized with plasmid vectors containing genes for F1, LcrV, with a gene for LT (heat-labile enterotoxin as an adjuvant) were protective against pneumonic plague^45^. In this report, mice were immunized with recombinant plasmids coated with 1.6-μm gold particles and shot with the gene gun on the ears. The animals were immunized on days 0 and 3 months before intranasal challenge after 8 months following the last boost with *Y. pestis* CO92^45^.

A DNA vaccine designed to protect against both anthrax and plague was evaluated in mice^119^. DNA constructs comprising fusions of V with a truncated anthrax lethal factor (LF) or LF with F1 or V alone, were coated to gold nanoparticles and delivered by gene gun to A/J mice and were shown to protect fully against challenge 21days later wth aerosolized *B. anthracis* and to 80% against aerosolised *Y. pestis*^119^.

#### mRNA

More recently, mRNA technology has been extended to produce candidate plague vaccines. An mRNA vaccine expressing a circularly permutated form of F1 delivered in lipid nanoparticles (LNP) protected (100%) of mice against bubonic disease after only a single dose^120^, whilst a self-amplifying mRNA LNP vaccine expressing both F1 and V was immunogenic in 2 doses and also protected outbred mice against a recent clinical isolate of *Y. pestis* from Madagascar in a bubonic plague model^121^. Both of these vaccines induced humoral and cell-mediated immune responses (Table 4) and show promise for future development, allowing a very flexible platform into which additional or modified RNA could be added, if needed. Furthermore, rapid advances in large scale manufacturing and formulation achieved for mRNA vaccines for SARS-CoV-2 are now readily transferrable to the plague application^122^.

### Translation to clinical development

Some vaccine approaches discussed above have transitioned to early clinical development (Fig. 2). Currently, a formulation of recombinant F1V (rF1V) in alum, supplemented with CpG 1018 is in Phase 2 clinical trial (Dynavax, USA)^3^. This form of CpG has already been incorporated in a Hepatitis B vaccine and approved by the FDA for clinical use in adults^123^. Also in phase 2 clinical trial is a formulation of native F1 with rV in alum (Lanzhou Institute and Jiangsu Provincial CDC, China)^3^. The third subunit vaccine currently in Phase 1 clinical trial comprises rF1V adjuvanted with flagellin (National Institute of Allergy and Infectious Diseases, USA)^3,124^.

The EV76-NIIEG vaccine is still approved only in China and Russia where it is in Phase 4 clinical trial. Since 2002, there have been vaccination campaigns with EV76 in 16-18 provinces of Mongolia by the National Centre for Zoonotic Diseases, Ulaanbaatar, Mongolia^3^. In a vaccination campaign report from plague-endemic foci in Mongolia, an adverse event rate of 7.3% with a 5.6% breakthrough in protection has been reported^3^. In October 2023, there was an ongoing campaign with EV76 in Mongolia in response to an outbreak of plague^3^.

### Strategies to gain evidence of vaccine efficacy

As with nearly all clinical prophylaxes or therapies, the pathway to regulatory approval is time-consuming, expensive and difficult, requiring evidence (direct, indirect or deduced) of human efficacy. This is especially challenging for vaccines against Tier-1 select agents such as *Y. pestis*, as human challenge studies are unethical. Further, due to the endemic nature of the disease, the number of infected patients is not large enough to draw meaningful conclusions on vaccine efficacy. Here we review the strategies available to demonstrate vaccine efficacy for plague.

### Animal data to support licensing

**Fig. 3:**
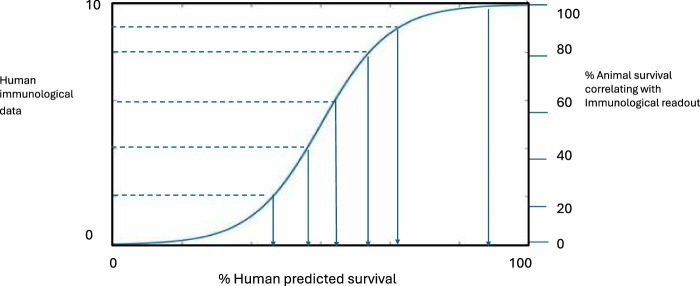
Immunobridging to predict vaccine efficacy in man. The figure depicts the use of percentage survival in vaccinated animals, which correlates with an immunological readout(s), to compare with the same immunological readout determined in a clinical trial, to predict vaccine efficacy in human.

Because of the pathogenicity of *Y. pestis*, its potential for epidemic spread, and the unpredictable nature and size of regional plague outbreaks, the feasibility of Phase 3 trials, whether preventive or reactive in nature, is under discussion^3^. Even in Madagascar, where the plague season is well known, the number of cases involved varies, so a Phase 3 trial may not be sufficiently powered, unless successive seasons are used. Pathways to licensure may therefore comprise the scrutiny of immunogenicity and efficacy data generated in animal models under Good Laboratory Practice (GLP) using the FDA’s Animal rule^125^ with the human immunogenicity data generated in clinical trials (*i.e*. immunobridging)^126^ (Fig. 3).

Evidence of efficacy could then be gained post-licensure. There is precedent for this with the Marketing Authorization under exceptional circumstances of an Ebola vaccine by the European Medicines Agency on the basis of human serum antibody data^127^. The FDA also has an accelerated approval pathway but cautions that the bar for approval of a vaccine for the pneumonic indication would be set higher than for the bubonic indication^3^.

### Immunobridging from animal models to human

The vaccine approaches reviewed here have predominantly been screened in mice (outbred as well as inbred)^128^, with some in Brown Norway rats^112,129^ and a few in NHPs^48,68,69,114,130^ all of which are authentic models for plague and evaluate both antibody and cell-mediated immunity^131^. The most consistent NHP model is the cynomolgus macaque^3,114^. However, increasing diversity in response occurs with escalation up to NHPs and the human population^132^. Table 5 summarises the studies which have been performed in NHPs to determine the efficacy of vaccines which are in preclinical development.

### Immune correlates of protection and surrogate markers of efficacy

Many researchers have now shown that antibody titers to the F1 and V proteins correlate with protection against bubonic and pneumonic plague in a range of animal models^66,68,69^, but the induction of cell-mediated immunity (CMI)^131,132^ and particularly a balanced Th1/Th2 response^56,93,103,133,134^ provides an optimal strategy for protection. The observation, *inter alia*, that mice immunized with the rF1-V vaccine and depleted of TNFα and IFNγ just prior to challenge, had poor survival compared with immunized controls which were not depleted^135^ indicated key roles for these Th1 cytokines in the development of protective immunity and these cumulative data have spurred the formulation of vaccine candidates which induce appropriately balanced immunity.

To enable effective immunobridging of animal data to human, it is preferable that researchers and developers use similar approaches to the measurement of antibody and cell-mediated responses^3,126,132^. The assay of specific antibody titers by quantitative ELISA (including the species-agnostic BRIDGE ELISA)^69^ is clearly important and provides a convenient surrogate marker of efficacy in the clinic.

Of equal importance is to assess cell-mediated immunity by ex vivo recall assay on animal tissue or human whole blood samples (by ELIspot or by flow cytometry)^8,103,136^ to determine the establishment of immune memory, and hence the need and spacing of booster doses. The ability also to assay for the induction of functional antibodies has been facilitated by the development of neutralizing monoclonal antibodies, particularly to V, enabling the development of competitive ELISAs^82,137–139^, which may be important aids in the down-selection of promising candidates in research. There is also ongoing effort to establish human reference serum for plague to provide an international standard as a reference point for serological surrogate markers of efficacy and thus to enable vaccine development^140^.

The WHO has published a draft target product profile (TPP) for a future plague vaccine, which sets out the qualifying criteria in terms of schedule, administration route, presentation, target efficacies in reactive and preventive modes, stability, and coverage, which would be applied to any plague vaccine candidate^141^.

### Future prospects

As highlighted in this review, there are some very promising vaccine candidates in the development pipeline with the potential to prevent plague in vulnerable populations. Here, we have also highlighted the epidemic potential of this disease and of *Y. pestis*, which in the absence of an approved vaccine, remains a serious biothreat. Seasonal outbreaks in Madagascar and other endemic regions cause fatalities every year. The potential for climate change to enhance this human vulnerability to plague in endemic regions or beyond is also being closely monoitored^142,143^. Climate change has already affected other zoonoses by extending the vector species to cause outbreaks of chikungunya and zika viruses in Central and South America^144,145^.

Aside from the difficulties of achieving statistically valid evidence of human efficacy, candidate vaccines may also fail because their manufacturing cannot be achieved at scale due to expense or feasibility. Thus, these promising candidate vaccines for plague are vulnerable to languish in the ‘valley of death’ without sustained and sufficient funding for clinical development and manufacturing at scale. Post COVID-19, the WHO has recently commented that ‘despite some recent progress, public heath vaccines are not available in all global regions and vaccines which have been prioritized by the WHO are not being developed or fully invested in, due to limited profit potential.’^146^ The Immunization Agenda 2030 endorsed by the WHO has a goal to reduce the incidence of, or to prevent, epidemics caused by vector-borne diseases by 2030^147^. These goals seem particularly relevant to plague prevention.

To date, there has been a significant investment in time and money in the research and early development of new plague vaccines. It is to be hoped that all the R&D activity on plague vaccines, which emerged after the decline in use of KWC vaccines, will be sustained and together with the regulatory pathways currently being mapped out, will lead to the approval of some new, safe, and fully efficacious vaccines to reduce disease prevalence. We recommend that global funding and health security systems take ambitious action to realize the potential of this investment in an approved vaccine(s) for plague.

## Supplementary information

Supplementary Information
