# Supplementary material for: Progress on the research and development of plague vaccines with a call to action
Source: NPJ Vaccines. 2024 Sep 7;9:162. doi: 10.1038/s41541-024-00958-1 (PMC11379892; doi:10.1038/s41541-024-00958-1)
Supplement: Supplementary file 1 — Supplementary Information [file 41541_2024_958_MOESM1_ESM.pdf]

OXFORD UNIVERSITY PRESS LICENSE
TERMS AND CONDITIONS

Mar 21, 2024

This Agreement between Dr. Ethel Williamson ("You") and Oxford University Press ("Oxford University Press") consists of your license details and the terms and conditions provided by Oxford University Press and Copyright Clearance Center.

License Number	5753521304382
License date	Mar 21, 2024
Licensed Content Publisher	Oxford University Press
Licensed Content Publication	Clinical & Experimental Immunology
Licensed Content Title	Vaccines for emerging pathogens: prospects for licensure
Licensed Content Author	Williamson, E D; Westlake, G E
Licensed Content Date	Apr 11, 2019
Licensed Content Volume	198

Licensed Content Issue	2
Type of Use	Journal
Requestor type	Author of this OUP content
Pharmaceutical support or sponsorship for this project	No
Format	Electronic
Portion	Figure/table
Number of figures/tables	1
Will you be translating?	No
Circulation/distribution	1
Title of new article	R&D of plague vaccines
Lead author	Ethel D. Williamson
Title of targeted journal	NPJ Vaccines

Publisher	Springer Nature
Expected publication date	Jun 2024
Order reference number	0324
Portions	Figure 2
Requestor Location	Dr. Ethel Williamson<br>Dairy Farmhouse<br>West Winterslow<br>Salisbury<br>Salisbury, Wiltshire SP5 1RE<br>United Kingdom<br>Attn: Dr. Ethel Williamson
Publisher Tax ID	GB125506730
Total	0.00 GBP

Terms and Conditions

STANDARD TERMS AND CONDITIONS FOR REPRODUCTION OF MATERIAL FROM AN OXFORD UNIVERSITY PRESS JOURNAL

1. Use of the material is restricted to the type of use specified in your order details.

2. This permission covers the use of the material in the **English** language in the following territory: *world*. If you have requested additional permission to translate this material, the terms and conditions of this reuse will be set out in clause 12.
